# Curative Resection for Thoracic Esophageal Carcinoma with an Aberrant Right Subclavian Artery: A Case Report

**DOI:** 10.70352/scrj.cr.26-0415

**Published:** 2026-10-01

**Authors:** Nobuyuki Nishida, Shinsuke Takeno, Makoto Ikenoue, Shun Munakata, Teru Chiyotanda, Fumiaki Kawano, Akiko Ichihara, Kazunosuke Yamada, Roko Hamada, Daisuke Hara, Kazuki Iwamoto, Kazuhiko Nakame, Makoto Matsukubo, Atsushi Nanashima

**Affiliations:** Department of Surgery, Faculty of Medicine, Miyazaki University, Miyazaki, Japan

**Keywords:** aberrant right subclavian artery, nonrecurrent inferior laryngeal nerve, esophageal cancer

## Abstract

**INTRODUCTION:**

Esophagectomy in the presence of an aberrant right subclavian artery (ARSA) is rare, and the anomalous vascular anatomy can complicate lymphadenectomy because of the risk of nerve injury.

**CASE PRESENTATION:**

The patient was a 67-year-old man who presented with pharyngeal discomfort. Right pyriform sinus cancer and middle thoracic esophageal cancer were detected. After neoadjuvant chemotherapy with docetaxel, cisplatin, and 5-fluorouracil for the esophageal cancer, the pyriform sinus cancer disappeared, indicating a complete response. Accordingly, esophagectomy with reconstruction using a gastric conduit was planned, with preservation of the hypopharynx. Because the presence of an ARSA had been recognized preoperatively, cervical lymphadenectomy was first performed via a cervical approach. Esophagectomy was performed thoracoscopically without injury to the nonrecurrent inferior laryngeal nerve (NRILN).

**CONCLUSIONS:**

Esophagectomy in a patient with an ARSA was performed while preserving the NRILN by initiating cervical lymphadenectomy before the thoracoscopic procedure.

## Abbreviations

ARSA
: aberrant right subclavian artery
Mt
: middle thoracic esophagus
NRILN
: nonrecurrent inferior laryngeal nerve
RLN
: recurrent laryngeal nerve
SCC
: squamous cell carcinoma
Ut
: upper thoracic esophagus

## INTRODUCTION

In the surgical treatment of thoracic esophageal cancer, bilateral lymph node dissection along the RLN is oncologically essential.^1)^ However, anatomical vascular anomalies can increase the risk of nerve injury. An ARSA is a rare congenital anomaly and is strongly associated with an NRILN.^2,3)^ In such cases, the atypical nerve course and a narrowed upper mediastinal space complicate safe lymph node dissection.^4)^

To overcome these challenges, cervical-first approaches and intraoperative nerve monitoring have been advocated.^5–7)^ We report a case of thoracic esophageal carcinoma with ARSA in which cervical lymphadenectomy was performed prior to thoracoscopic esophagectomy in the semi-left lateral decubitus position, allowing safe nerve preservation and oncologically adequate lymph node dissection.

## CASE PRESENTATION

The patient was a 67-year-old man who presented with pharyngeal discomfort. Upper gastrointestinal endoscopy revealed right pyriform sinus carcinoma and middle thoracic esophageal cancer, and histopathology confirmed SCC in both lesions. Contrast-enhanced CT demonstrated enlargement of the para-esophageal and bilateral RLN lymph nodes.

The patient was diagnosed with right pyriform sinus carcinoma (cT2, N2b, M0, SCC, cStage IVa) and thoracic esophageal carcinoma (Ut–Mt, 6.5 cm, type 2, SCC, cT3br, N1, M0, cStage IIIB). The initial plan was to perform total pharyngo-laryngo-esophagectomy following neoadjuvant chemotherapy with docetaxel, cisplatin, and 5-fluorouracil.

After neoadjuvant chemotherapy, the thoracic esophageal tumor showed marked shrinkage, and the pyriform sinus lesion achieved a complete response (**Fig. 1**). In consultation with the otolaryngology department, hypopharyngeal preservation was considered feasible, and chemoradiotherapy was subsequently planned for the pyriform sinus carcinoma. For the esophageal cancer, chemoradiotherapy was also presented as an alternative option; however, the patient strongly preferred surgical resection, and thoracoscopic esophagectomy was performed.

**Fig. 1:**
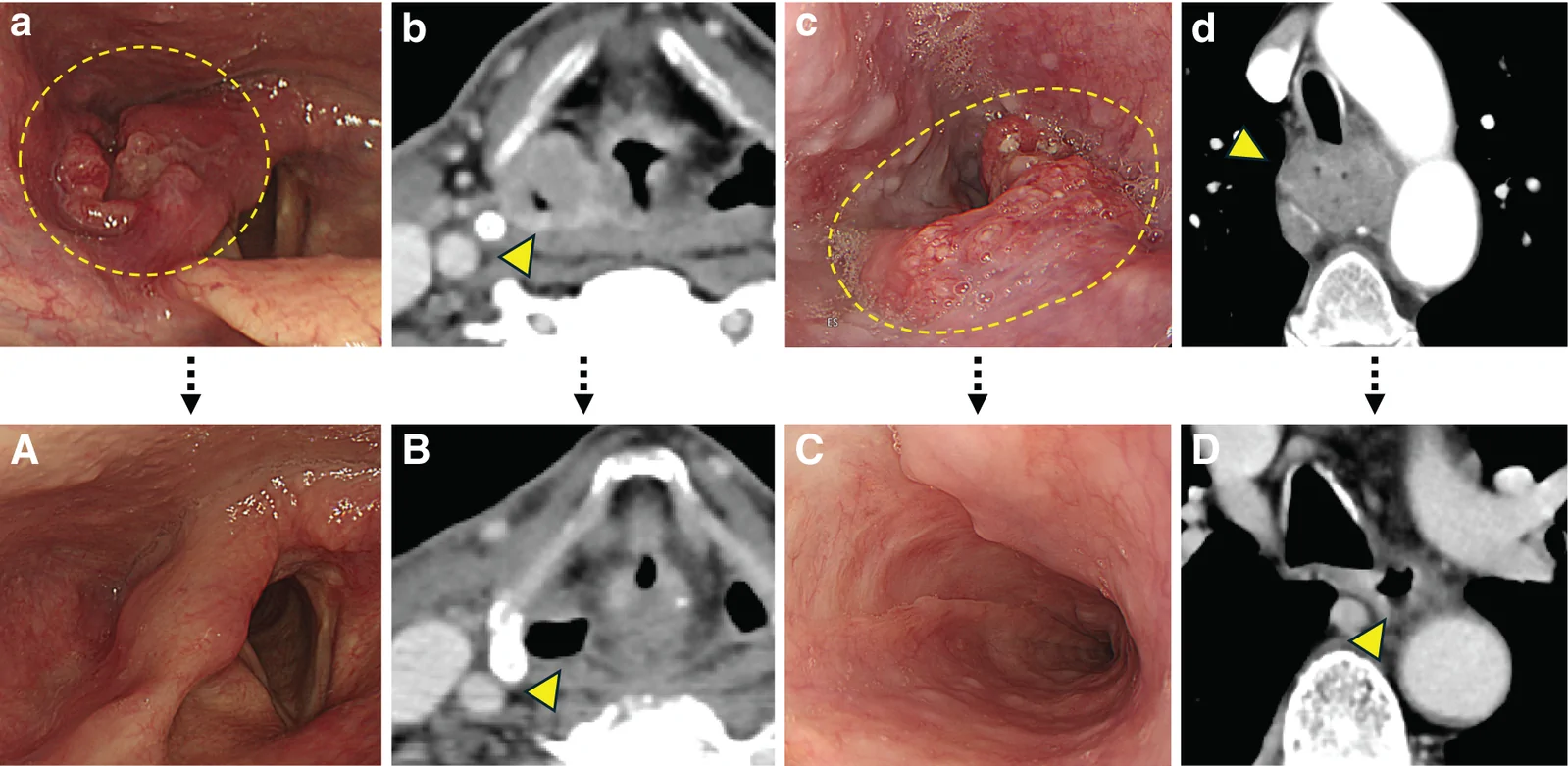
Findings after 3 courses of DCF chemotherapy. The area outlined in dashed lines and arrowheads indicate tumorous lesions. Tumorous lesions of the pyriform sinus and mid-thoracic esophagus (**a**, **b**, **c**, **d**). After 3 courses of DCF, the lesion in the right pyriform sinus showed marked regression and became indistinct on CT (**A**, **B**). The esophageal wall thickening was markedly reduced, with partial scar formation (**C**, **D**). DCF, docetaxel, cisplatin, and 5-fluorouracil

Because the presence of an ARSA had been recognized preoperatively (**Fig. 2**), cervical lymph node dissection was initiated first. Before starting the dissection, intraoperative nerve monitoring was used to identify the right vagus nerve and NRILN (**Fig. 3**).

**Fig. 2:**
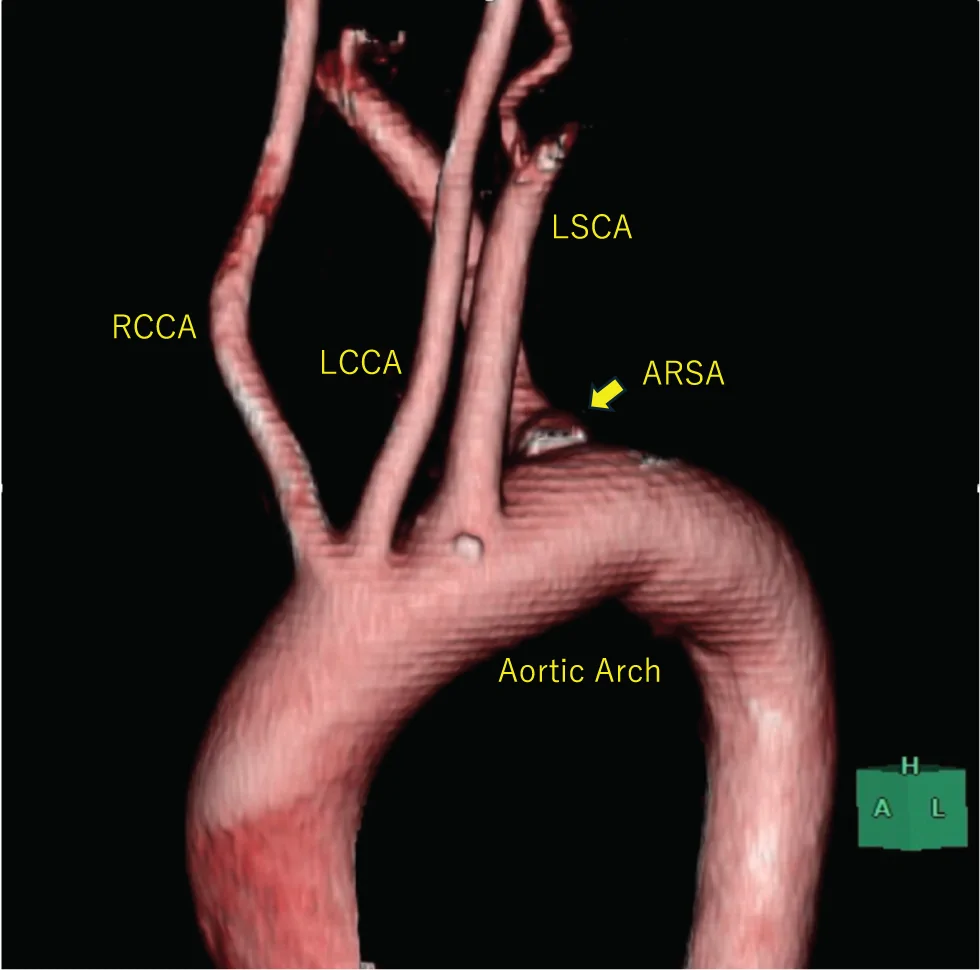
Preoperative 3D CTA. Preoperative 3D CTA revealed an ARSA arising as the last branch of the aortic arch. ARSA, aberrant right subclavian artery; LCCA, left common carotid artery; LSCA, left subclavian artery; RCCA, right common carotid artery

**Fig. 3:**
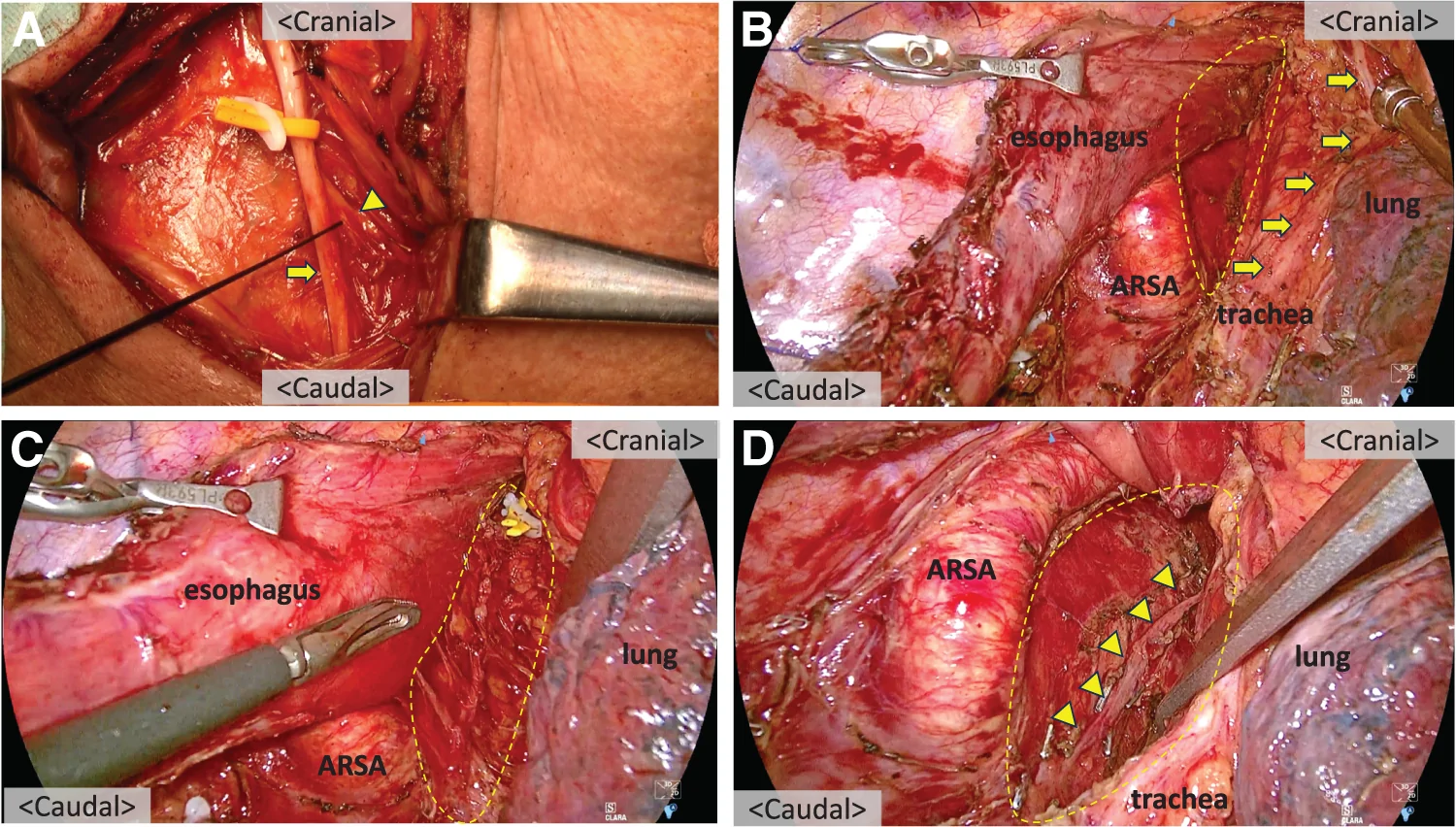
Intraoperative findings. The area outlined in dashed lines indicate the working space. (**A**) Cervical-first approach: Intraoperative view showing the right vagus nerve (arrow) and the NRILN (arrowhead), which were clearly identified and preserved using intraoperative nerve monitoring. (**B**–**D**) Thoracoscopic approach: Upper mediastinal lymph node dissection. The esophagus lies between the ARSA and the trachea. Lymph node dissection was performed in the limited operative field using the marker placed during the cervical procedure as a landmark. The arrows indicate the right vagus nerve, and the arrowheads indicate the left RLN. ARSA, aberrant right subclavian artery; NRILN, nonrecurrent inferior laryngeal nerve; RLN, recurrent laryngeal nerve

The operative time was 792 minutes, and the estimated blood loss was 0 mL. Intraoperative nerve monitoring was used to confirm the absence of RLN palsy. No postoperative RLN palsy was observed.

Although the patient developed pneumonia on POD 4 and required antibiotic treatment, the postoperative course was otherwise uneventful, and the patient was discharged home on POD 23. After a pathologic examination, no residual tumor cells were identified in either the resection margins or the lesion. The final pathological diagnosis was ypT0, N0 (0/44), M0, pathological complete response (Grade 3). The patient has remained disease-free for 16 months after treatment.

## DISCUSSION

ARSA is a rare congenital vascular anomaly, reported in approximately 0.5%–1.6% of the general population.^1)^ Although ARSA itself is usually asymptomatic, it has important surgical implications because it is strongly associated with NRILN.^2)^ In thoracic esophageal cancer, lymph node dissection along the RLN is oncologically essential, with reported metastasis rates of up to 40%.^3)^ Therefore, achieving both safe nerve preservation and adequate lymphadenectomy represents a major challenge in patients with ARSA.

Embryologically, ARSA results from regression of the right fourth aortic arch, leading to the absence of the normal looping structure around which the right RLN normally descends. Consequently, the nerve runs directly from the vagus nerve to the larynx as an NRILN. Previous studies have demonstrated that more than 80%–90% of patients with ARSA have an associated NRILN.^2,4)^ Thus, identification of ARSA on preoperative contrast-enhanced CT strongly suggests the presence of an NRILN and necessitates modification of the surgical strategy.

Thoracoscopic esophagectomy in patients with ARSA is technically demanding because the aberrant artery often narrows the upper mediastinal space and alters conventional anatomical landmarks. In addition, ARSA has been reported to be occasionally accompanied by thoracic duct anomalies, which may further complicate lymph node dissection along the RLN.^5)^ In the present case, however, no abnormal thoracic duct course was observed intraoperatively, allowing standard lymphadenectomy to be performed with careful anatomical confirmation.

Nevertheless, rare but fatal complications such as arterioesophageal fistula involving ARSA have been reported after esophagectomy,^6)^ highlighting the importance of meticulous dissection and careful operative planning.

We searched PubMed on June 13, 2026 for reports published from 2006 to 2026 using the terms “aberrant right subclavian artery” OR “ARSA” and “esophageal cancer” OR “esophageal carcinoma,” targeting reports that provided detailed descriptions of thoracoscopic surgery (**Table 1**).^7–13)^ In this search, we found no report that described in detail the use of a cervical-first approach in conjunction with thoracoscopic esophagectomy in the semi-left lateral decubitus position.

**Table 1:** Reported cases of VATS esophagectomy for esophageal cancer with ARSA (2006–2026, n = 11)

Case no.	Reference	Reported year	Age	Sex	Tumor location	Surgical procedure	Reconstruction route	Reconstruction organ	Final stage	Cervical-first approach	Operative time (min)
1	Kasashima^7)^	2014	73	Female	Middle thoracic (Mt)	VATS esophagectomy	Retrosternal route	Stomach	I B	No	NM
2	Kasashima^7)^	2014	63	Male	Lower thoracic (Lt)	VATS esophagectomy	Retrosternal route	Stomach	I A	Yes	NM
3	Shindo^8)^	2017	53	Male	Middle thoracic (Mt)	VATS esophagectomy	Posterior mediastinal route	Stomach	I A	NM	450
4	Shindo^8)^	2017	75	Male	Middle-Upper thoracic (MtUt)	VATS esophagectomy	Posterior mediastinal route	Stomach	III A	NM	722
5	Shindo^8)^	2017	72	Male	Middle thoracic (Mt)	VATS esophagectomy	Posterior mediastinal route	Stomach	III B	NM	582
6	Hashimoto^9)^	2020	85	Male	Lower thoracic (Lt)	Laparoscopic/mediastinoscopic esophagectomy	Posterior mediastinal route	Stomach	NM	No	338
7	Koyama^10)^	2022	70	Male	Middle thoracic (Mt)	VATS esophagectomy	Retrosternal route	Stomach	I	No	365
8	Liu^11)^	2025	30	Male	Middle-Lower thoracic (MtLt)	VATS esophagectomy	NM	Stomach	NM	NM	NM
9	Takeda^12)^	2025	72	Female	Lower thoracic (Lt)	VATS esophagectomy	Retrosternal route	Stomach	I	Yes	717
10	Mochizuki^13)^	2025	66	Male	Lower thoracic (Lt)	VATS esophagectomy	Retrosternal route	Stomach	I	Yes	727
11	Present case	2026	67	Male	Middle thoracic (Mt)	VATS esophagectomy	Subcutaneous route	Stomach	0	Yes	792

ARSA, aberrant right subclavian artery; NM, not mentioned; VATS, video-assisted thoracoscopic surgery

Several reports have described the usefulness of cervical-first approaches and intraoperative nerve monitoring in esophagectomy for patients with ARSA.^13–15)^ In the present case, we started with the cervical phase, during which cervicothoracic lymph node dissection was performed as extensively as possible. This cervical-first strategy enabled early identification and preservation of both the right-sided NRILN and the left RLN under direct vision, thereby enhancing the safety and completeness of the subsequent thoracic dissection.

Although vascular anatomy can be accurately evaluated preoperatively using CT, the course of the laryngeal nerves is difficult to predict. Therefore, the operation was planned according to the possible anatomical variations of the NRILN described by Toniato et al.^4)^ Intraoperatively, the nerve was identified using intraoperative nerve monitoring, and meticulous sharp and blunt dissection around any cord-like structure was prioritized while avoiding the use of energy devices until the nerve had been clearly identified.

During the thoracic phase, upper mediastinal lymphadenectomy was performed by tracing the vagus nerve cranially after prior identification of the NRILN during the cervical procedure. This approach may reduce both the psychological burden on the surgeon and the risk of inadvertent nerve injury.

The ARSA follows a retroesophageal course in approximately 80%–84% of cases.^16)^ This anatomical feature often poses a challenge during upper mediastinal lymph node dissection (**Fig. 4**). In the present case, the ARSA coursed posterior to the esophagus, with the trachea interposed between these structures, enabling the preoperative prediction of technically demanding dissection around the left RLN lymph node station. Performing the cervical phase first established the cranial dissection plane before the thoracic procedure, thereby facilitating subsequent thoracoscopic lymphadenectomy and reducing the technical difficulty of upper mediastinal dissection.

**Fig. 4:**
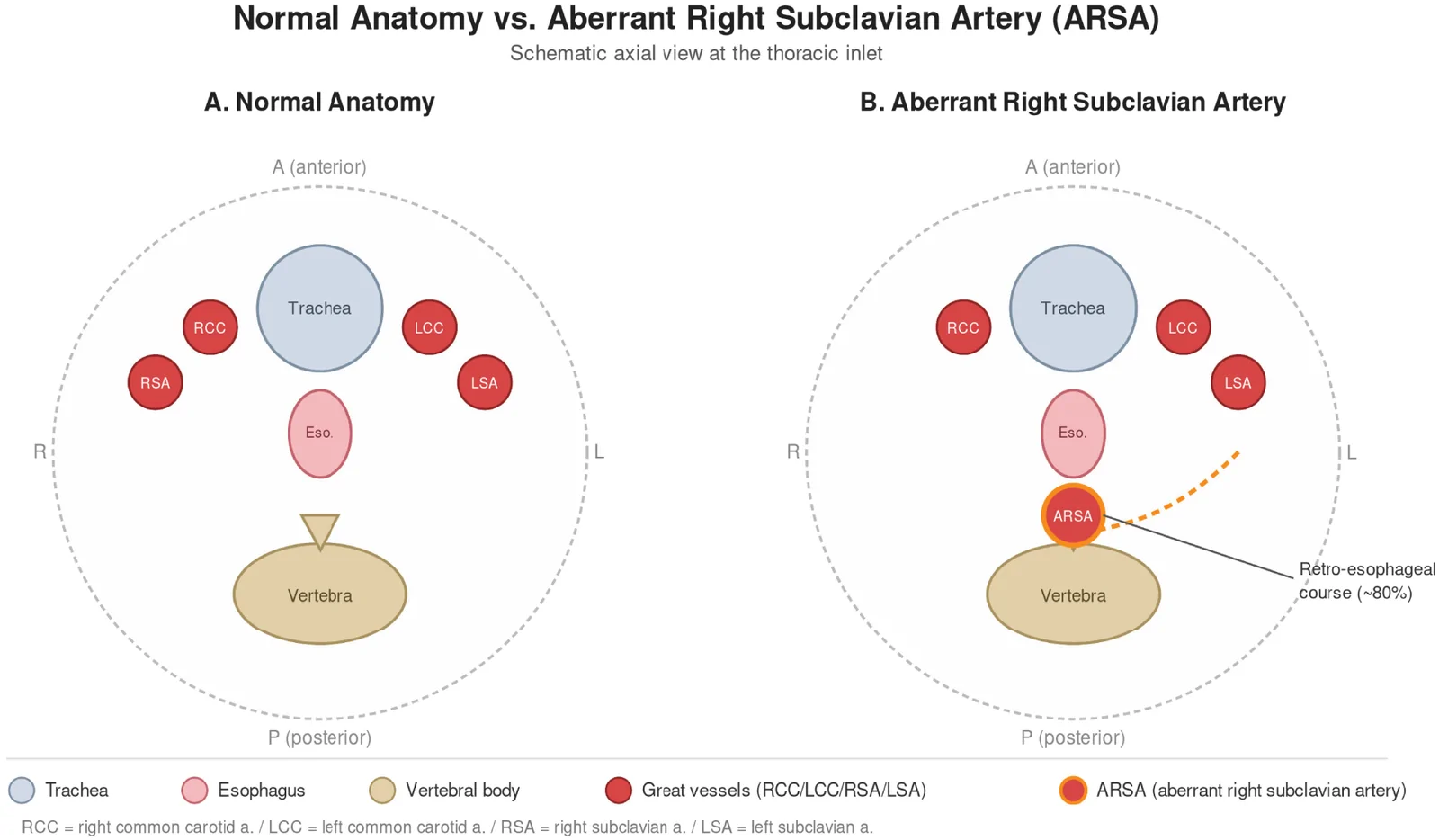
Normal anatomy and an ARSA. The ARSA most commonly follows a retroesophageal course, which often poses a challenge during upper mediastinal lymph node dissection. ARSA, aberrant right subclavian artery

While prone positioning has been reported to provide a stable thoracoscopic view, the semi-left lateral decubitus position offers specific advantages, particularly for lymph node dissection along the RLN. By rotating the operating table, gravity-assisted lung retraction during middle and lower mediastinal dissection provides an operative field comparable to that achieved in the prone position. During upper mediastinal dissection, however, lung position can be adjusted more flexibly, facilitating precise dissection around the cervicothoracic junction. Moreover, this position allows a dorsal-to-ventral approach to the RLN, which is advantageous for safe and oncologically adequate dissection of the RLN lymph nodes while maintaining continuous awareness of nerve anatomy.

Otsuka et al. introduced the native tissue preservation technique, which preserves the RLN in its anatomical position during lymphadenectomy and may contribute to reducing postoperative RLN palsy.^17)^ In addition, during upper mediastinal lymphadenectomy, the course of the vagus nerve is aligned with the surgeon’s hand movements, enabling more delicate and atraumatic dissection around the nerve. This technical advantage may be particularly beneficial in patients with anatomical anomalies and in cases requiring upper mediastinal dissection within a limited working space.

## CONCLUSIONS

Esophagectomy in patients with ARSA requires careful preoperative evaluation and a tailored operative strategy. A cervical-first approach that enables extensive bilateral cervicothoracic lymph node dissection, combined with thoracoscopic esophagectomy in the semi-left lateral decubitus position, offers clear advantages for safe and oncologically adequate lymphadenectomy while minimizing the risk of nerve injury.

## DECLARATIONS

### Availability of data and materials

This article is a case report, and no datasets were generated or analyzed during the current study. Therefore, this section is not applicable.
